# Editorial: Bioimaging applications in biosensors and biomolecular electronics

**DOI:** 10.3389/fbioe.2025.1568124

**Published:** 2025-04-25

**Authors:** Sai Kumar Tammina, Abuelmagd Mahmoud Abdelmonem, Naveen Kumar Reddy Bogireddy

**Affiliations:** ^1^ Department of Chemical and Petroleum Engineering, Khalifa University of Science and Technology (KUST), Abu Dhabi, United Arab Emirates; ^2^ Food Technology Research Institute (FTRI), Agricultural Research Center (ARC), Giza, Egypt; ^3^ Chemistry Department, Royal College of Surgeons in Ireland (RCSI), Dublin, Ireland; ^4^ Instituto de Ciencias Físicas, National Autonomous University of Mexico (UNAM), México City, Mexico

**Keywords:** biosensors, electronics, biomaterials, bio-inspired, biomimetic, biorobotics, smart, stimuli-responsive

This editorial summarizes the contributions to the Frontiers Research Topic “Bioimaging Applications in Biosensors and Biomolecular Electronics,” established under the Biosensors and Biomolecular Electronics and appearing under the Frontiers in Bioengineering and Biotechnology journals. After more than a decade of extensive investigation of bio-fabricated materials, they have evolved into one of our most valuable and intriguing nanomaterials. The current state of scientific studies on bionanomaterials, with a shift from standard procedures to multifunctional hybrids, is a testament to the field’s continuous advancement, published annually. These materials, with their potential applications in the environment, energy, and health, are a testament to the versatility of biomaterials. The interest in biomaterials is driven by various factors, including their ease and affordability to synthesize using eco-friendly or sustainable procedures, readily accessible surface functionalization, and their capacity to form complexes with other species. These unique physical properties, improved performance, and excellent stability make bionanomaterials stand out (Kaur et al., 2023). The evolution in developing and understanding biosensing and biomolecular systems with sustainable materials procedures has increased the scientific community’s interest in implementing such systems for all analytes (Dhall et al., 2024; Bhatia et al., 2024). However, the need for advanced biosensing technology is urgent, and further research and development in this area are crucial.

**FIGURE 1:**
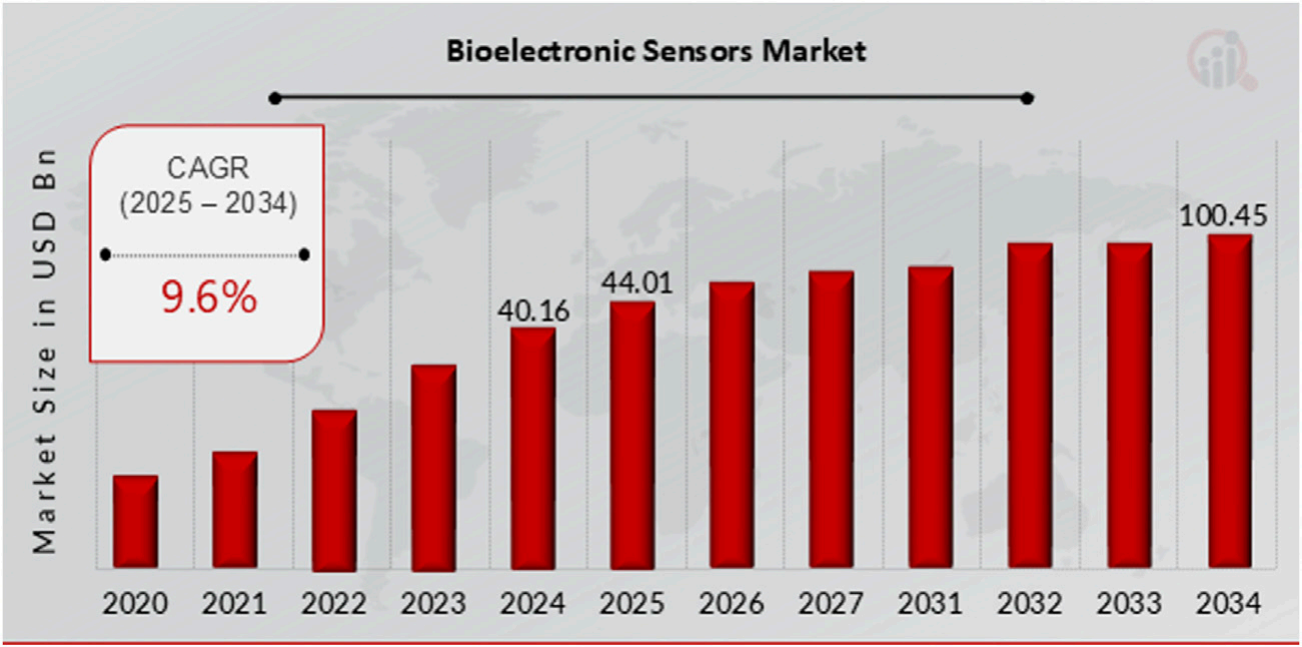
Secondary research, primary research, MRFR database and analyst review (Dhall et al., 2024).

By 2023, the global biosensors market was projected to reach a staggering USD 30.1 billion, with a promising compound annual growth rate (CAGR) of 7.7%–9.6% from 2024 to 2034 (Figure 1). This rapid market growth, fueled by innovative and next-generation biosensor technology, presents a thrilling landscape for the future. The demand for point-of-care testing propels the development of tiny electrochemical and microfluidic immunosensors. Because advanced biosensors can give precise, real-time measurements of biological and chemical substances, they have a wide range of applications in many different sectors. For instance, medical diagnostics delivered disease detection kits for glucose sensors for diabetes, tumor markers for cancer, and pathogen detection for contagious diseases. Also, wearable health devices monitor heart rate, sweat composition, oxygen saturation, and body temperature. Increasing investments in wearable medical device R&D for female fertility (Ankit, 2025; Global Statistics Report – 2032, 2023) further underscore this market growth potential for researchers and industry professionals in bionanomaterials. Moreover, many startups and established companies contribute to developing advanced wearables for chronic disease diagnosis and real-time monitoring. Their innovative technologies make insulin-dependent diabetic testing devices easier, more portable, and more convenient, increasing acceptance and improving patients’ quality of life (Ankit, 2025).

So, there is vast potential for fabricating highly efficient biomaterials with broad spectral emissions, improved yield efficiencies, and easy-to-use, reusable probes. Beyond the current understanding of the formation of biomaterials, there is a pressing need to delve deeper into the source behind the atomic and molecular-level engineering-based selectivity of a specific analyte. This ongoing scientific exploration is crucial for unlocking the full potential of bionanomaterials (Sharma et al., 2025).

Opportunities exist to enhance the design of biomaterials for future applications at the field level. This involves addressing issues such as collateral contamination from biomaterial probes and improving efficiency in reproducibility, recovery, purification, and separation from the reaction solution. However, the true scientific breakthrough lies in understanding the formation and interaction mechanisms, a goal that can be achieved through the design and execution of well-organized experimental designs. The selectivity of the developed sensor for a particular target is always challenging, so researchers must explore novel biosensor architectures with extremely selective sensitivity, even in complex settings. Especially when dealing with complicated biological samples (such as blood or urine), biosensors may encounter difficulties because of interference with other components in the sample.

Also, to attain very low detection limits and advance the possibility for practical application in therapeutic domains, future research should also focus more on developing highly sensitive sensing devices on targeted cells and infections. Also, certain biosensors could take a long time to give precise readings, which could be a drawback for applications that must be completed quickly. Additionally, the restricted solubility of bionanomaterials in bodily fluids has hindered their use in biological sensing and imaging applications, presenting a significant challenge that requires further research and innovation to overcome. In addition to neural/cardiac recording, the creation of extremely biocompatible, electrochemically active, and flexible implantable tissue monitors and neural electrodes using sophisticated microfabrication and 3D printing methods for real-time monitoring of the cellular or tissue signals is crucial for the advancement of prosthetic devices, artificial intelligence, and robots in the future. However, producing and maintaining high-quality biosensors may be costly, which might prevent their widespread usage. Thus, developing biosensors at a limited and affordable price can demand the widespread production and utility of biosensors.

On the other hand, nanomaterials with their unique physical and chemical properties (e.g., high surface area, size-tunable optical properties, superparamagnetism, and ease of functionalization) have emerged in the field of bioimaging, offering unparalleled advantages in different aspects (sensitivity, resolution, and multifunctionality) from molecular level to whole-body imaging. For instance, nanoparticles with unique photophysical (e.g., photostability and tunable emission) properties and superparamagnetic nanoparticles have proven highly valuable in long-term tracking and multiplex bioimaging. Furthermore, by tailoring, surface modification, and functionalizing the nanoparticles with responsive coatings or biomolecules, researchers can develop targeted imaging probes that selectively accumulate at disease sites, improving diagnostic accuracy. Integrating nanomaterials of different functionalities (plasmonic, fluorescent, magnetic) into multimodal and/or multifunctional advanced materials further enhances their applications in bioimaging, treatment-targeted delivery, and controlled release (Gao et al., 2020; Encarnación et al., 2022; Han et al., 2019).
